# Surface-Initiated Atom Transfer Radical Polymerization for the Preparation of Well-Defined Organic–Inorganic Hybrid Nanomaterials

**DOI:** 10.3390/ma12183030

**Published:** 2019-09-18

**Authors:** Monika Flejszar, Paweł Chmielarz

**Affiliations:** Department of Physical Chemistry, Faculty of Chemistry, Rzeszow University of Technology, Al. Powstańców Warszawy 6, 35-959 Rzeszów, Poland

**Keywords:** nanoparticles, surface-initiated atom transfer polymerization, organic–inorganic hybrid nanomaterials

## Abstract

Surface-initiated atom transfer radical polymerization (SI-ATRP) is a powerful tool that allows for the synthesis of organic–inorganic hybrid nanomaterials with high potential applications in many disciplines. This review presents synthetic achievements and modifications of nanoparticles *via* SI-ATRP described in literature last decade. The work mainly focuses on the research development of silica, gold and iron polymer-grafted nanoparticles as well as nature-based materials like nanocellulose. Moreover, typical single examples of nanoparticles modification, i.e., ZnO, are presented. The organic–inorganic hybrid systems received according to the reversible deactivation radical polymerization (RDRP) approach with drastically reduced catalyst complex concentration indicate a wide range of applications of materials including biomedicine and microelectronic devices.

## 1. Introduction

The crucial moment coming with the development of reversible deactivation radical polymerization (RDRP) methods was the invention of the atom transfer radical polymerization (ATRP) technique [1,2,3,4]. ATRP is one of the most widely used configuration of RDRP solutions, based on adequate equilibrium between active radicals and dormant species [5,6,7,8,9]. In the next stage, propagating radicals in the presence of monomers are deactivated and returned to dormant state. The controlled nature of ATRP allows to obtains polymers that are distinguished by a narrow molecular weight distribution (MWD) and diverse molecular architecture [10,11,12,13,14,15,16,17]. In this context, precise control over molecular weight (MW), composition and brush thickness provides interesting opportunity to synthesis a variety of functional (co)polymer brushes [18,19]. Subsequent advantage in polymer synthesis *via* ATRP methods is a library of compatible monomers including mono- and polyfunctional acrylates [20,21,22], α-substituted monomers [23,24] and also acrylamides [25,26,27], which makes this method attractive for the preparation of a wide range of commercially available polymers and hybrid materials.

**Scheme 1:**
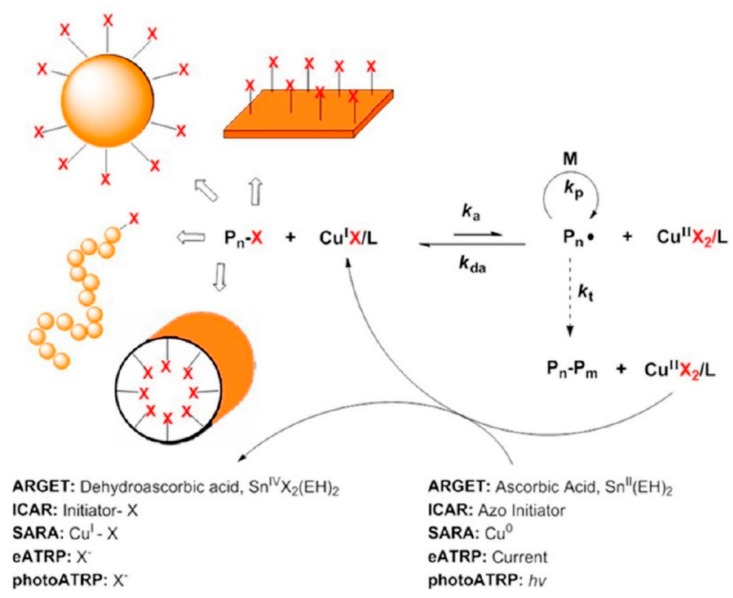
Schematic illustration for ATRP and surface-initiated atom transfer radical polymerization (SI-ATRP) technique. Reproduced with permission from reference [29].

In ATRP reactions, the presence of the transition metal catalytic complex (generally Cu^I^X/L) plays a crucial role [5]. The dormant species (alkyl halides) react reversibly with catalyst–metal complexes in lower oxidation state Cu^I^X/L causing the formation of reactive radicals (P_n_^●^) and the transition of metal complex with coordinated metal ligand to higher oxidation stage (Cu^II^X_2_/L) [27,28]. The process of a single or several monomer addition is deactivated by the reaction, in which the growing radicals are converted to dormant species and in the same time a catalytic complex in the lower oxidation stage is regenerated (Scheme 1) [21,29].

Hybrid polymer materials are based on the covalent linkage of the polymer chain to the various inorganic substrates including nanoparticles (NPs), flat and cylindrical surfaces or the inner surface of the nanopores [30]. Such surface modification can be achieved *via* different techniques including (a) *grafting-from* [31,32,33,34,35]; (b) *grafting-through* [36,37,38]; (c) *grafting-onto* [39,40,41]. 

**Scheme 2:**
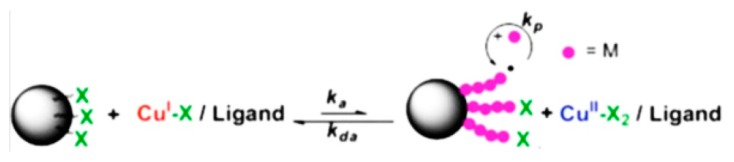
Schematic illustration for “grafting-from” approach employed to introduce polymer brushes onto the surface of a nanoparticle *via* SI-ATRP. Reproduced with permission from reference [30], 2015 Springer International Publishing Switzerland.

The *grafting-from* method uses the presence of a polymerization initiator connected by a covalent bond to the substrate surface enabling the formation of polymer chains on inorganic surfaces. Additionally, the initiating group may be an integral part of the polymer chain, or could be attached so as to functionalize on the surface. Control over the architecture, density, functionality and thickness of a grafted polymer chain is the main advantage of the *grafting-from* approach. In addition, this method can be applied to surfaces of various geometries including flat surfaces, nanoparticles and porous materials, as well as compositions of complex metals, metal oxides and natural compounds [42,43,44]. Ultimately, it leads to the production of hybrid materials with high grafting density [30,35]. Scheme 2 presents the reaction scheme involving “*grafting-from*” method. 

**Scheme 3:**
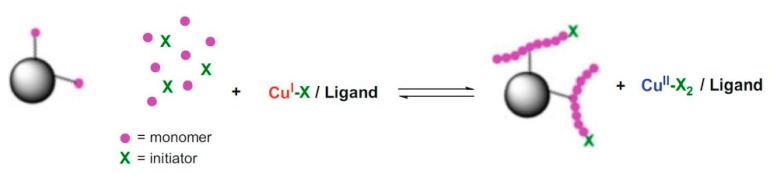
Schematic illustration for “grafting-through” approach for introduction of polymer brushes to the surface of NPs using SI-ATRP. Reproduced with permission from reference [30], 2015 Springer International Publishing Switzerland.

In the second technique, *grafting-through*, the first step includes an addition of a polymerized monomer unit to the modified surface. Subsequently, the created macromonomer is copolymerized with low molecular weight monomers. This approach enables the synthesis of nanomaterials using inorganic macromonomers as well as natural products and other polymers [45,46]. The ratio of monomer and macromonomer concentrations used in the synthesis determines the density of polymer chains grafting, but in most cases *grafting-through* approach enables obtaining lower density brushes than *grafting-from* or *grafting-onto,* therefore it is less frequently used. Scheme 3 presents the reaction scheme for “*grafting-through*” method.

**Scheme 4:**
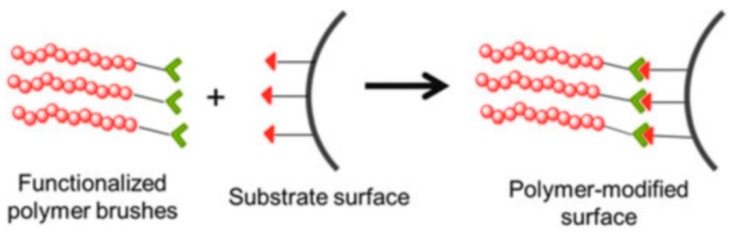
Schematic illustration for “grafting onto” approach using “click” chemistry. Reproduced with permission from reference [30], 2015 Springer International Publishing Switzerland.

The *grafting-onto* approach is based on reaction between functionalized polymer brushes and surface substrate including associated functional groups compatible to end-functional group in polymer chains. The steric hindrance and slow diffusion of polymer molecule to substrate surface partially limit employment of this method in the synthesis of polymer hybrid. However, “click chemistry” successfully applies *grafting-onto* to linking polymer chains with functionalized surface in reaction *via* Cu^I^-catalyzed azide–alkine cycloaddition [47,48]. A significant advantage of the *grafting-onto* approach over other approaches is the possibility of prior physical and chemical characterization of the polymer to be attached. Then, the result is a well-defined material with no complicated synthetic procedure (Scheme 4) [49,50].

The ability to synthesize polymer brushes on the surface of nanoparticles (e.g., gold (Au) [51,52], silver (Ag) [53], silica (Si) [35,54], titanium (Ti) [55], cobalt (Co) [56], and zinc (Zn) [39]) *via* the SI-ATRP approach provides efficient tool that can be applied in a wide range of engineering applications such as a membrane technology [57] as well as biomedical applications [56,58,59,60] including immunotherapy [61] and antifouling surfaces [62]. 

## 2. Gold Nanoparticles

The organic polymer layer covering the metallic core of the gold nanoparticles leads to the creation of hybrid materials, which can be used as a molecular diagnostic tool as well as element of the drug delivery system, hence the wide interest in the modification of such nanoparticles exhibiting high application ability in biomedical fields. It is worth noting that intensified research on the modification of gold nanoparticles in recent years effected in simple modifications [63] through a core–shell structure of gold NPs [64] or even advanced biomedical applications such as the SiRNA delivery system reported in 2018 [52].

**Scheme 5:**
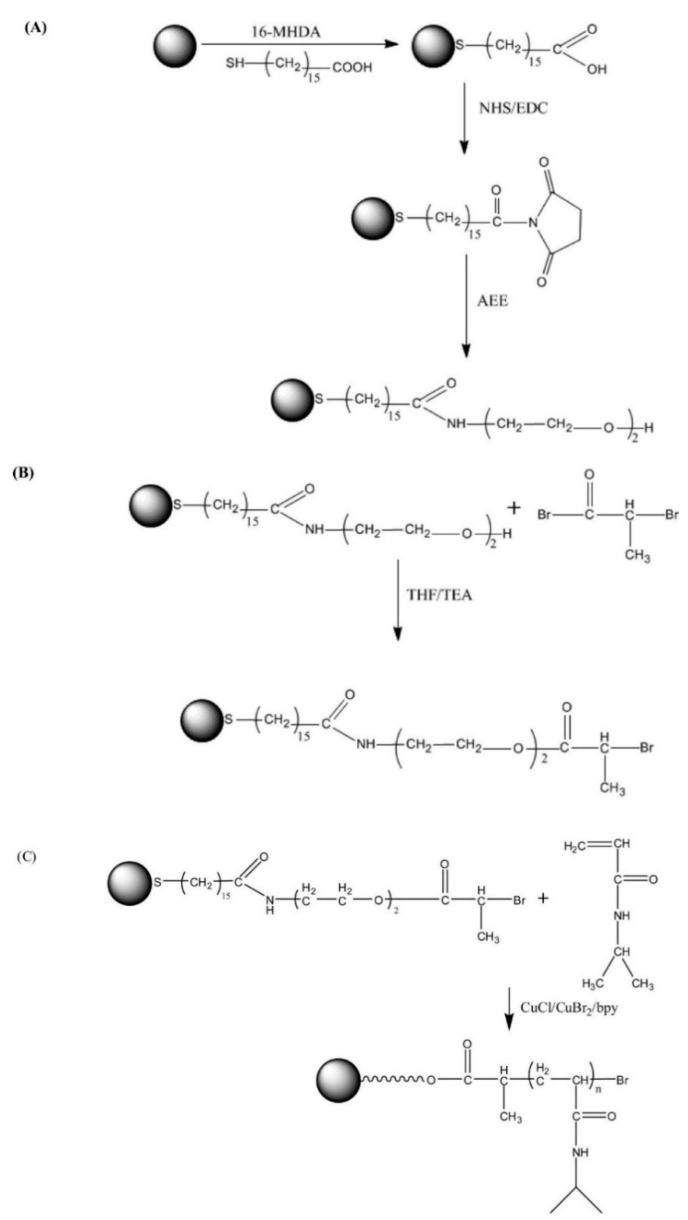
Schematic illustration for the surface modification of gold nanoparticles. (**A**) Coupling of AEE to functionalized gold nanoparticles resulting in creation of a terminal hydroxyl group. (**B**) Preparation of initiator based on gold nanoparticles. (**C**) Polymerization of NIPAM from Au NPs through SI-ATRP approach. Reproduced with permission from reference [63].

In 2010, Chakraborty et al. grafted poly(*N*-isopropylacrylamide) (PNIPAM) from Au NPs *via* SI-ATRP using 2-bromopropionyl bromide (2-bpb) as the initiator. The polymerization was carried out under environmentally friendly conditions with an aqueous solution at room temperature. In the first step of the performed modification, 2-(2-aminoethoxy)ethanol (AEE) was coupled to carboxyl-functionalized gold nanoparticles using *N*-(3-dimethylaminopropyl)-*N′*-ethylcarbodiimide hydrochloride/*N*-hydroxysuccinimide (EDC/NHS), resulting in the terminal group being a hydroxyl group (Scheme 5A). In the second stage, the hydroxyl-terminated gold nanoparticles reacted with the 2-bpb initiator in the presence of tetrahydrofuran/triethylamine (THF/TEA) (Scheme 5B). Finally, the NPs were inoculated with an initiator and after that were exposed to *N*-isopropylacrylamide (NIPAM) in the presence of a catalytic complex (Cu^I^Cl/Cu^II^Br_2_/2,2′-bipyridyl (bpy)) to undergo polymerization to form nanoparticles grafted with PNIPAM (Scheme 5C) [63]. 

Recently, Song et al. [64] presented dependence between the shape of polymeric gold hybrid nanoparticles versus the ratio between initiator and the Au NPs used in SI-ATRP with 917 ppm of Cu catalyst species (Cu^I^Br/*N*,*N*,*N*′,*N*″,*N*″-pentamethyldiethylenetriamine (PMDETA)). The core–shell or asymmetric structures has been prepared through adjusting the proportion of disulfide initiator and gold nanoparticles. 

**Figure 1:**
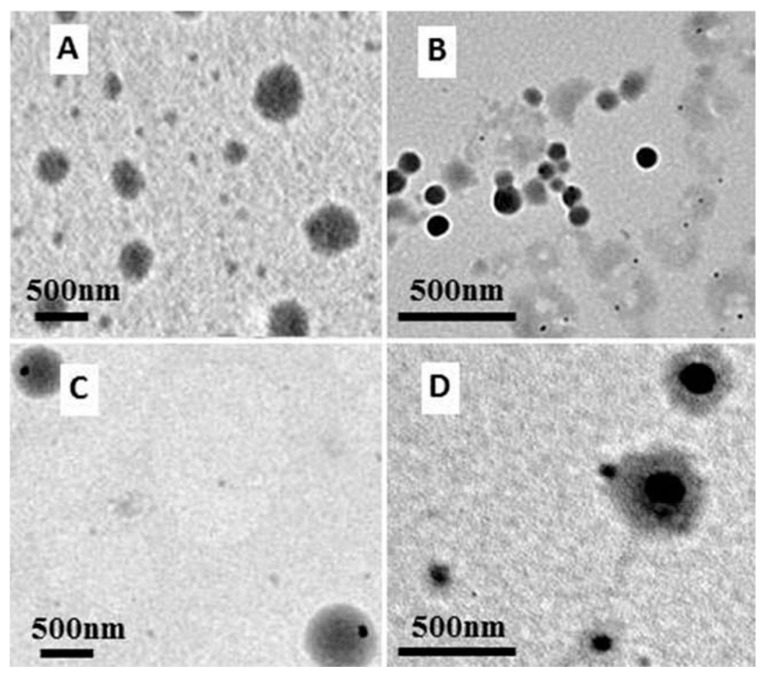
TEM pictures of gold-polystyrene hybrid nanoparticle (molar ratio of Au NPs and disulfide initiator: (**A**) 1/100; (**B**) 1/1000; (**C**) 1/10,000; (**D**) 1/20,000. Reproduced with permission from reference [64].

In order to simplify the experimental design and reduce the toxicity of used solvents such as THF or *N*,*N*-dimethylformamide (DMF), gold nanoparticles were transferred to the organic phase using ammonium bromide tetraoctyl (TAOB) as a phase transfer agent. The polymerization reaction of styrene was carried out in cyclohexane at 70 °C for 4 h in an inert gas atmosphere. The amount of the initiator on the nanoparticle surface affected the thickness of the grafted brushes and the formation of new hybrid structures. Transmission electron microscope (TEM) pictures showed that if 0.025–0.25 mL of disulfide initiator was added to the gold nanoparticles, an asymmetric structure of polystyrene–gold hybrid was obtained and a single gold nanoparticle was attached with a polystyrene sphere (Figure 1). If the disulfide initiator was in excess, the particles of the core–shell polymer–metal nanohybrid material was synthesized as a gold nanoparticle encapsulated by a uniform polymeric coating [64].

**Scheme 6:**
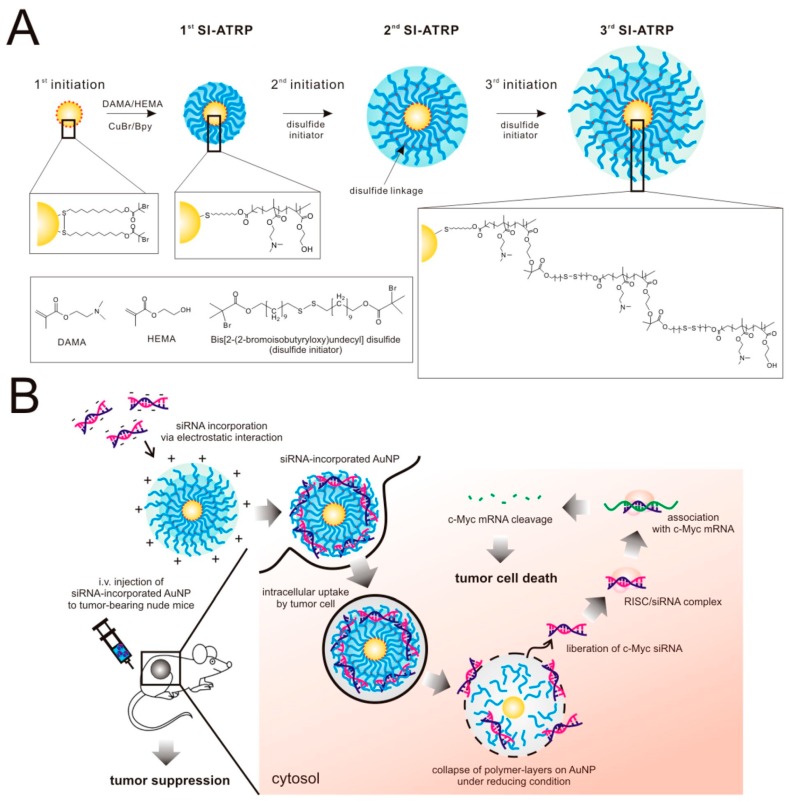
(**A**) The scheme of preparing polymer multilayered gold nanoparticles *via* surface-initiated ATRP. (**B**) Schematic illustration for proposed anti-cancer effect of c-Myc siRNA incorporated AuNP and cellular uptake process. Reproduced with permission from reference [52], 2017, American Chemical Society.

More recently, Kim et al. [52] reported an interesting SiRNA delivery system involving gold nanoparticles modified *via* surface initiated ATRP technique with 10,000 ppm of Cu^I^Br/bpy. The first stage includes the synthesis of polymeric multiply coatings using monomers such as (diethylamine)ethyl methacrylate (DAMA) and 2-hydroxyethyl methacrylate (HEMA). Then SiRNAs molecules were electrostatically incorporated with Au NPs cationic coatings, where the charge of the attached SiRNA is controlled by various number of coatings. In order to produce multiple coatings on gold nanoparticles, a disulfide initiator was used to connect polymeric layers and disintegrated under cytoplasmic conditions (Scheme 6). Intravenous delivery of siRNA anti-c-Myc for an A549 tumor xenograft murine model showed a significant tumor regression [52].

## 3. Silica Nanoparticles

Undoubtedly, silica nanoparticles are the most common and most widely used nanoparticles modified *via* the SI-ATRP method with various monomers including methyl acrylate (MA) [65], methyl methacrylate (MMA) [66], 2-hydroxyethyl acrylate (HEA) [67], styrene [68,69], 2-(dimethylamino)ethyl methacrylate [70], γ-methacryloxypropyltrimethoxysilane (PMPTS) [71], (*N*,*N*-dimethylaminoethyl methacrylate (DMAEMA) [72,73,74,75], poly(ethylene glycol) methacrylate [76,77], NIPAM [78,79,80,81], *tert*-butyl acrylate (*t*BA) [35,82], di(ethylene glycol) methyl ether methacrylate (DEGMA) [83] and other monomers [54,80,84,85,86,87]. These hybrid nanomaterials combine the advantages of nanoparticles including its rigidity and stability and organic polymer layers characterized by flexibility, processability and functionality to enlarge the potential applications such nanohybrids. Recent literature reports show that the grafting of polymer brushes from the surface of Si nanoparticles was applied, among others, to produce cross-linked polymer nanocapsules [65,67], coatings with excellent anti-icing properties [85] and protein-resistance characteristic [77] as well as pH-responsive hybrid Janus colloids [75].

### 3.1. Superhydrophobic Nanocomposite Material Based on Silica Nanoparticles

Wettability of flat surfaces is a phenomenon that plays an important role both in nature and in industrial applications [88]. Surface roughness and chemical interactions occurring on a flat surface determine the wetting angle characterizing the appropriate material. In the last decade, superhydrophobic surfaces with a wetting angle greater than 100° and even above 150° for materials with rough or hierarchical nanostructure attract increasing attention [89,90,91]. Therefore, numerous attempts to obtain superphydrophobic systems using silica nanoparticles modified with SI-ATRP method can be found in the literature [68,71,85,92].

**Scheme 7:**
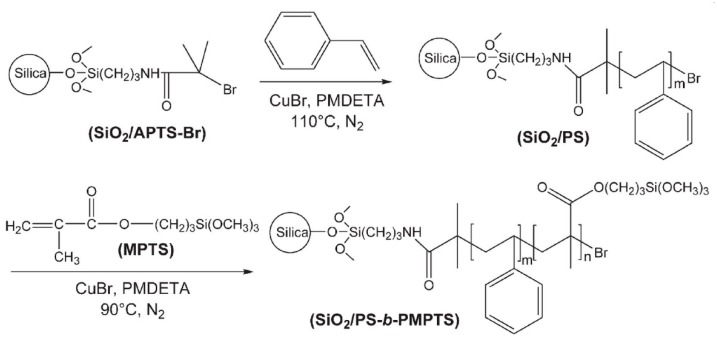
Schematic illustration for formation of SiO_2_/PS-*b*-PMPTS nanoparticles *via* SI-ATRP. Reproduced with permission from reference [68].

The controlled manner of the synthesis of organic–inorganic hydrophobic hybrid nanoparticles modified with polystyrene (SiO_2_/PS) and polystyrene-*b*-poly(methacryloxypropyltrimethoxysilane) (SiO_2_/PS-*b*-PMPTS) was presented by Yu et al. [68]. They synthesized SiO_2_/PS and SiO_2_/PS-*b*-PMPTS platforms using initiator-modified SiO_2_ particles (SiO_2_/APTS-Br) *via* SI-ATRP with 20,000 ppm of Cu catalyst species (Scheme 7). 

Scanning electron microscope (SEM) photographs taken for silica nanoparticles before and after grafting of polymer brushes clearly indicated the presence of a homogeneous polymer coating on the surface of the metallic core. The uniformly distributed silica nanoparticles observed under the microscope differed in their microstructure and surface roughness. For nanoparticles modified with PS-*b*-PMPTS copolymer, a fine-grained surface consisting of microphases separated from each other was observed. The particle size of SiO_2_/PS and SiO_2_/PS-*b*-PMPTS estimated from SEM measurements showed around 45 and 90 nm, respectively. The wetting angle value determined for SiO_2_/PS was 153° and for SiO_2_/PS-*b*-PMPTS 143°, both obtained hybrid systems showed superhydrophobic properties dependent on the combination of roughness and surface chemistry. 

**Scheme 8:**
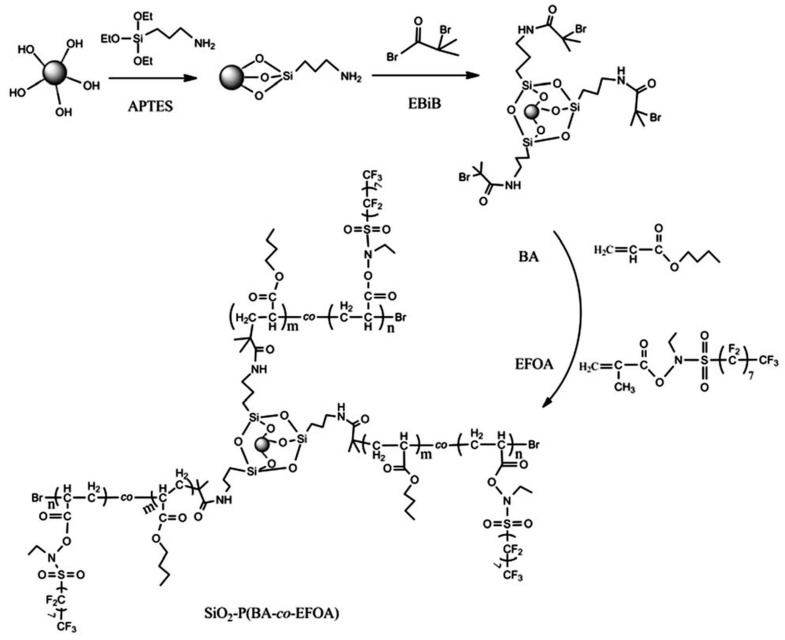
Schematic illustration of general approaches to prepare the amino-functionalized, 2-bromoisobutyrate-functionalized and poly(BA-*co*-EFOA) grafted silica particles. Reproduced with permission from reference [85].

More recently, Zhan et al. [85] described a superhydrophobic hybrid nanocomposite with excellent anti-icing properties synthesized *via* activators generated by electron transfer (AGET) ATRP with 9090 ppm of Cu^II^Br_2_/PMDETA [85]. *n*-Butyl acrylate (BA) and 2-(*N*-ethyl-perfluorooctanesulphamido) acrylate (EFOA) were grafted from amino-functionalized silica nanoparticles SiO_2_–NH–Br using ethyl α-bromoisobutyrate (EBiB) as an efficient initiator. The pristine silica nanoparticles was previously functionalized by triethylamine and 2-bromoisobutyrate according to Scheme 8. 

**Figure 2:**
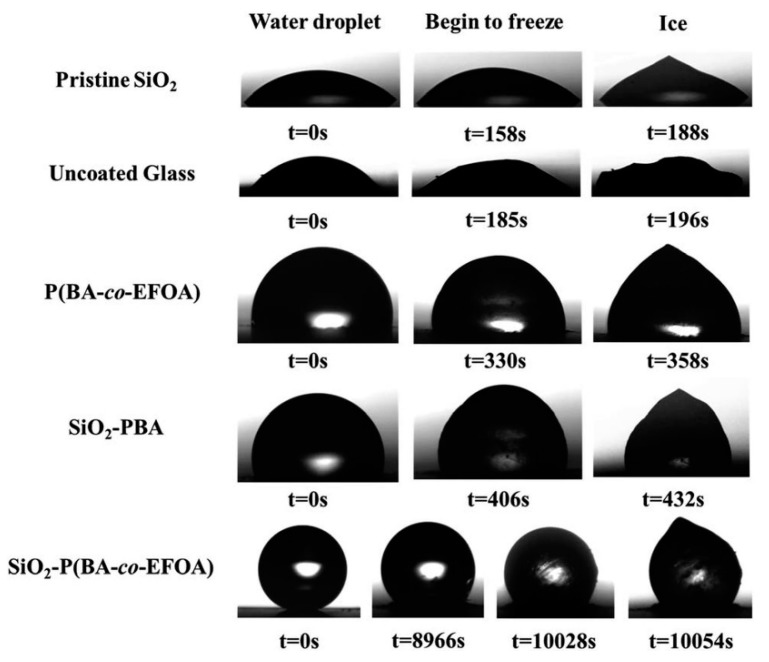
Photographs of individual water droplets on glass with different coatings during the whole freezing process captured by a high-speed CCD camera. Reproduced with permission from reference [85].

The anti-icing properties of the synthesized material were confirmed by contact angle measurement showing the extremely high value (170°) for nanoparticles covered with a layer of copolymer. Moreover, the delay in crystallization of condensed water was observed. The copolymer coating not only promotes the removal of droplets but also delays the freezing time (Figure 2). The time needed to freeze a drop of water on a surface with a temperature of −18 °C was 10,054 s. The P(BA-*co*-EFOA) polymer layer on the nanoparticle surface forms a kind of adiabatic layer that contributes to the reduction of crystallization temperature by 6.82 °C and guarantees a high application potential of functionalized NPs.

Silicon nitride (SiN_4_) can also be used as the metallic core of organic–inorganic hybrid nanosystems using silica nanoparticles. The material used in ceramics, due to its abrasion resistance and thermal resistance, was subjected to functionalization and then the brushes of poly(methyl methacrylate) (PMMA) were grafted from the surface of SiN_4_ NPs [92]. Si_3_N_4_ nanoparticles covered with a layer of polymer brushes are characterized by better dispersibility and stability in organic solvents than unmodified nanoparticles. Consequently, it is possible to select the type of grafted polymer brushes depending on the potential application of the nanomaterial [92].

### 3.2. Stimuli-Responsive Hybrid Materials Based on Silica NPs

Intelligent nanodevices created by the development of modern microelectronics require the application of advanced materials such as silicon wafers or silica NPs modified with a layer of stimulated-responsive polymer brushes. Therefore, efficient way of obtaining modern materials responding to external stimuli are intensively sought. The most frequently proposed solution is grafting from the surface of NPs (co)polymer brushes containing thermo-sensitive PNIPAM as a building segment [78,79,80,81].

In 2012, thermo-responsive amphiphilic Janus Si NPs were received *via* a combination of “polymer single crystal templating” and “*grafting-from”* approach [78]. In the first step, silica nanoparticles with a diameter of 40–50 nm were immobilized on the surface of single crystals. Then on the uncovered part of Si NPs PNIPAM brushes were grafted using SI-ATRP methodology with Cu^II^Br_2_/tris [2-(dimethylamino) ethyl] amine (Me_6_TREN) (12,000–12,050 ppm) as a catalytic complex. After dissolution of single-polymer crystals, Janus NPs with bicompartment poly(ε-caprolactone) (PCL) and PNIPAM brushes were received.

**Scheme 9:**
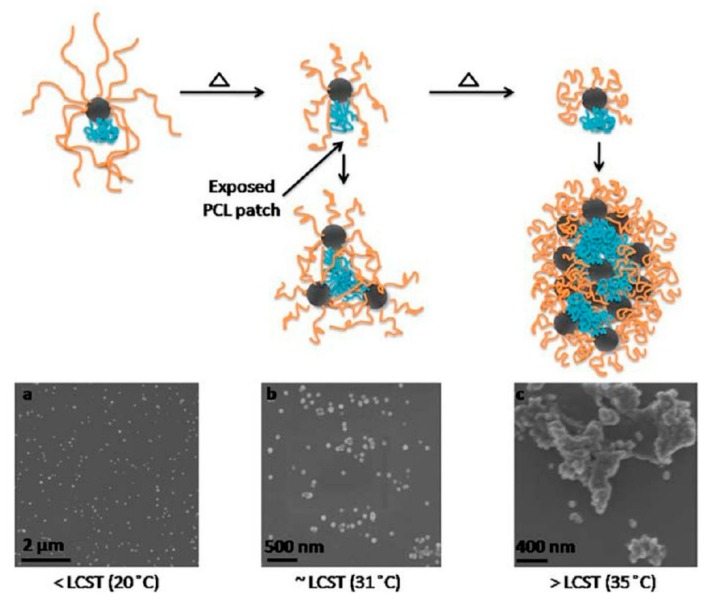
Schematic illustration of LCST behavior of PCL-SiNPs-PNIPAM Janus particles in aqueous solution and SEM images (**a**–**c**) of the assembled structures of Janus nanoparticles in solution at corresponding temperatures. Reproduced with permission from reference [78].

Using staining methods and a TEM microscope, the asymmetrical character of the synthesized material was demonstrated. Moreover, comparison of Janus NPs with NPs modified with PNIPAM brushes only showed that lower transition temperature and narrower transition range were observed for Janus NPs. As a result of the presence of PCL chains on the surface of nanoparticles, hydrophobic interactions leading to much greater driving force for the agglomeration of Janus particles within the temperature range of lower critical solution temperature (LCST) (Scheme 9) [78].

More recently, the synthesis of silica nanoparticles modified with thermo-sensitive PNIPAM brushes to produce inorganic-organic microspheres with core/shell structure (SPM) was described [79]. Then SPM was dispersed in aqueous solution and packed as photonic crystals (PC) in a dry state, thereafter using optical microscopy the packing behavior of photonic crystals was observed.

By increasing the temperature above the LCST of PNIPAM, reversible swelling and shrinkage of the PNIPAM coating causes dispersion and the precipitation of SPM in aqueous solution. When the temperature rises above the LCST, the microdispersed PCs were packed tightly. Changing distances as the temperature rises above LCST, it changed color from red to blue, which could have resulted in to be watched with the naked eye for incident angle. Therefore, such materials have high application potential to be used as a visual temperature sensor [79].

Interestingly, Wu et al. [80] have synthesized hybrid silica nanoparticles densely-grafted with dually-responsive macromolecular brushes *via* SI-ATRP, for perspective application to construction of smart nanodevices e.g., in nanoelectronic solutions. 

**Scheme 10:**
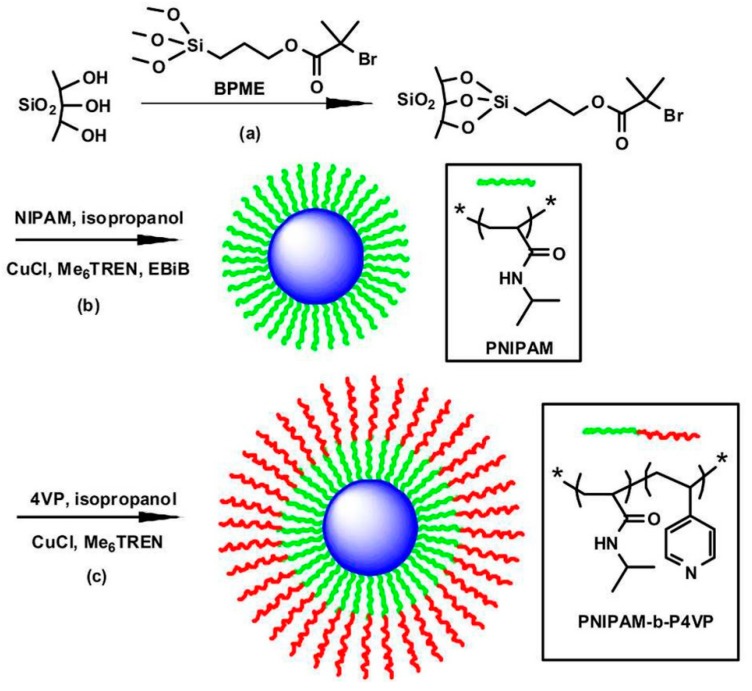
Schematic illustration of synthetic procedure to receive SiNPs-*g*-PNIPAM-*b*-P4VP: (**a**) Immobilization of ATRP initiator, 3-(2-bromo-2-methyl) propionyloxylpropyl] trimethoxysilane (BPME), onto SNPs; (**b**) Synthesis of SNPs-*g*-PNIPAM *via* surface-initiated ATRP; and (**c**) Synthesis of SNPs-*g*-PNIPAM-*b*-P4VP by chain extension from SNPs-*g*-PNIPAM with 4VP. Reproduced with permission from reference [80].

In the first step of facile synthesis of thermo and pH dual-responsive poly(*N*-isopropylacrylamide)-*b*-poly(4-vinylpyridine)-grafted silica nanoparticles (SiNPs-*g*-PNIPAM-*b*-P4VP) an ATRP initiator-functionalized silane, was attached onto the surfaces of Si NPs to initiate ATRP (4350–12,580 ppm of Cu^I^Cl/Me_6_TREN) of thermo-sensitive NIPAM from the nanoparticle surface (Scheme 10). Respectively, halogen exchange and sacrificial initiator were introduced to ensure the controlled manner of polymerization process [80].

The original idea of generation and thermally adjustable catalysis of silver nanoparticle immobilized temperature-sensitive silicon nanocomposite and mild reducing reactions was proposed by Xu et al. [81]. Firstly, PNIPAM was grafted from Si NPs surface *via* ATRP with 12,500 ppm of catalyst spices (Cu^I^Br/PMDETA), and then the mild reduction reaction led to the anchoring of Ag nanoparticles on the surface of the polymer layer covering the metallic core of the synthesized system.

The structural hybrid composite consisting of silica nanoparticles coated with the PNIPAM-Ag coating showed good dispersing properties. Furthermore, the thermo-sensitive properties of the polymeric coating enable the control of the amount of aggregated Ag catalyst on the polymer carrier. The resulting system serves as a catalytic system for the degradation of various nitrobenzenes and organic pigments in an aqueous solution with sodium borohydride (NaBH_4_) at ambient temperature. Hence, the presented material can be used in sensing devices and particular in water purification as well as green chemistry [81].

Recently, Penelas et al. have developed bioinert core-brush hybrid nanoparticles modified with thermosensitive DEGMA for future applications in biosensor, tissue engineering and optical system [83].

**Scheme 11:**
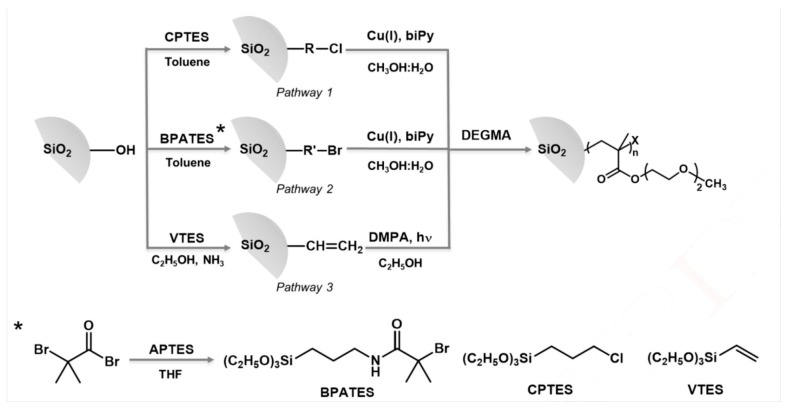
Schematic illustration of surface-initiated ATRP of DEGMA on SiO_2_-Cl NPs. Reproduced with permission from reference [83]. VTES—vinyltriethoxysilane.

Three different synthetic approaches have been presented that have successfully led to PEG-functionalized colloids (Scheme 11). Two types of chloropropyltriethoxysilane (CPTES) and (3-aminopropyl) triethoxysilane (APTS) initiators were immobilized on the surface of nanoparticles and used to perform surface initiated controlled radical polymerization (ATRP with 20,000 ppm of catalyst complex). Then, PDEGMA brushes were grafted from previously modified nanoparticles SiO_2_-Br and SiO_2_-Cl. Moreover, photo-grafting is the third equally effective synthetic route for (SiO_2_-V)-*g*-PDEGMA NPs preparation [83].

### 3.3. Amphiphilic Polymer Coatings in Preparation of Hybrid Nanocomposites via SI-ATRP

Various approaches to find the optimal and efficient way to obtain stabilized colloidal solutions of nanoparticles for optics, electronic or biomedicine devices were taking into consideration [93,94,95]. In addition to ligand stabilization or surface silanization, another way is to use amphiphilic monomers to synthesize polymer coatings stabilizing nanoparticles in aqueous solution. This subsection presents the latest selected achievements in the synthesis of amphiphilic polymer coatings by surface-initiated ATRP.

The novel strategy to create crosslinking nanocapsules with the use of surface initiated ATRP approach was published by the Liu research group [65,67] from Lanzhou University in 2009 and 2010. 

**Scheme 12:**
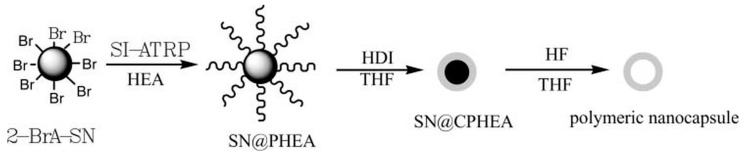
Schematic illustration of the preparation process of crosslinked poly(2-hydroxyethyl acrylate) (PHEA) nanocapsules. Reproduced with permission from reference [67].

The first attempt to obtain nanocapsules *via* SI-ATRP with 13,600 ppm of Cu^I^Br/bpy from previously a modified silica nanotemplate was made with the use of poly(methyl acrylate) (PMA) [65] then, following the direction of biomedical applications and the design of drug delivery systems, the biocompatible PHEA (10,030 ppm of Cu^I^Br/bpy) according to Scheme 12 was used [67]. After the modification of hydroxyl side-groups of silica NPs by PHEA polymer brushes was crosslinked with hexamethylene diisocyanate (HDI), subsequently the silica templates encapsulated in the crosslinked polymer shells were removed by etching with HF to create the precisely designed nanocapsules with a diameter *ca.* 20–50 nm [67].

**Scheme 13:**
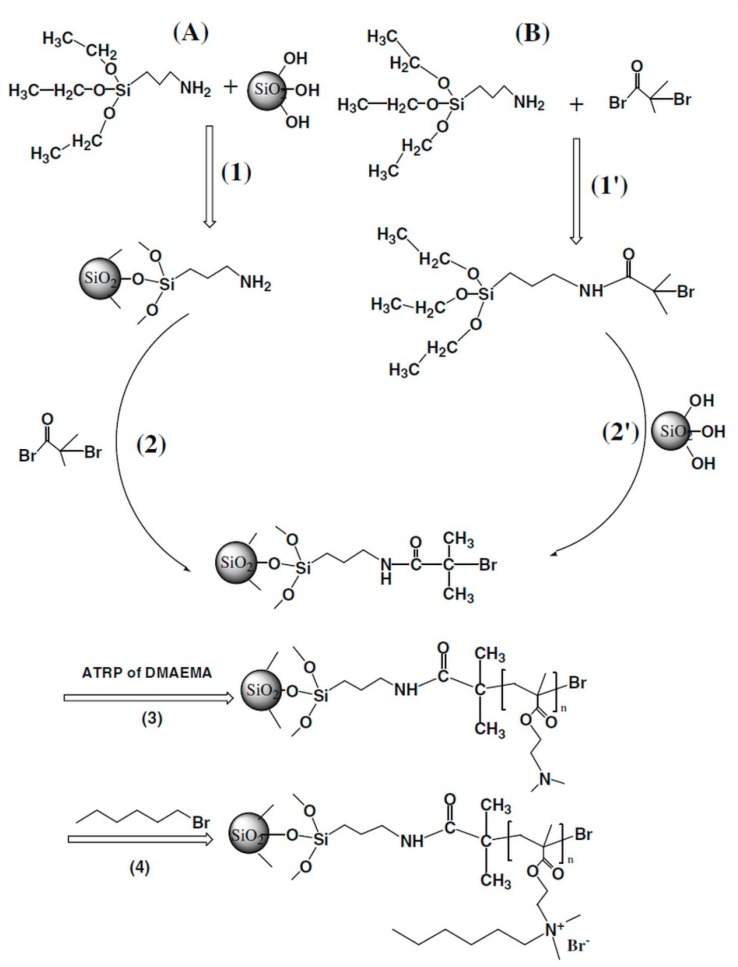
Schematic illustration of synthesis route of SiO_2_-*g*-P(DMAEMA-Q) nanocomposites (Q denote quaternization of PDMAEMA) using 2-bromoisobutyratefunctionalized silica NPs prepared *via* two approaches including (**A**) modification of SiO_2_-NH_2_ nanoparticles in the presence of toluene and triethylamine, followed by BiBB (**B**) preparation of macroinitiators of SiO_2_ coated with APTES-BIBB. Reproduced with permission from reference [70].

Recently, Wang et al. prepared well-defined densely grafted poly(2-(dimethylamino) ethyl methacrylate) (PDMAEMA) brushes grafted from silica nanoparticles [70]. Synthesis of 2-bromoisobutyrate-functionalized silica nanoparticles was performed *via* two different approaches (Scheme 13). At first, SiO_2_-NH_2_ nanoparticles, toluene and triethylamine, were placed in a flask and then α-bromoisobutyryl bromide (BIBB) was added dropwise. The reaction was carried out at 0 °C for 30 min and then at room temperature for 24 h. The second approach was based on synthesis of macroinitiators of SiO_2_ coated with γ-aminopropyltriethoxysilane-α-bromoisobutyryl bromide (APTES-BIBB). Thus, SiO_2_ was mixed with dry toluene to produce a homogeneous suspension and then APTES-BIBB was added. The synthesis was realized *via* ATRP with 4070 ppm of catalyst complex (Cu^I^Br/PMDETA) [70].

**Figure 3:**
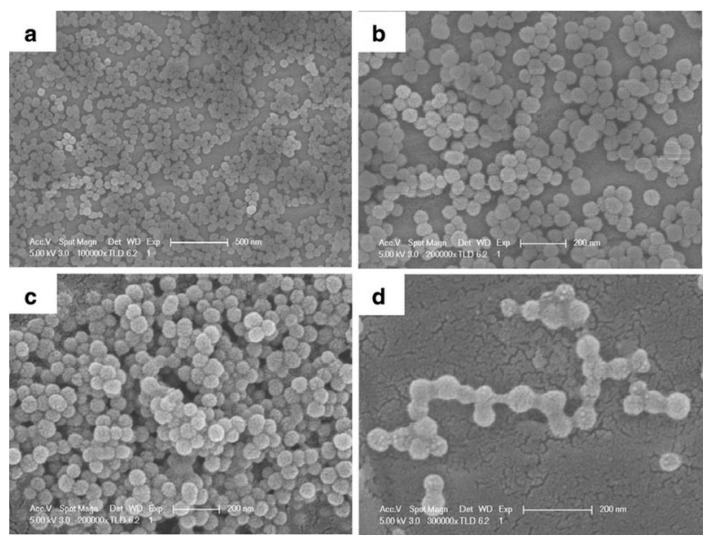
SEM images of pristine SiO_2_ (**a**,**b**) and SiO_2_-*g*-P(DMAEMA-Q) (**c**,**d**). Reproduced with permission from reference [70].

Spherical shape of the resulting nanocomposite with core-shell morphology and diameter of about 50 nm was confirmed by SEM technique (Figure 3) [70].

**Scheme 14:**
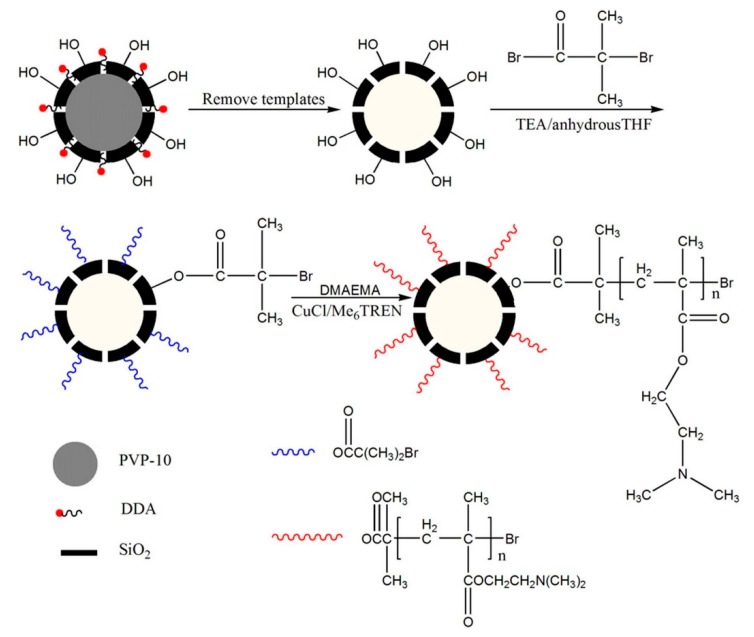
Schematic illustration of preparation of PDMAEMA-modified HMSN. Reproduced with permission from reference [72].

Meanwhile, Yu [72] synthesized PDMAEMA on the surface of hollow mesoporous silica nanoparticles (HMSN) by SI-ATRP technique with 6670 ppm of Cu^I^Cl/Me_6_TREN complex (Scheme 14). 

Poly(*N*,*N*-dimethylaminoethyl methacrylate) as a thermal- and pH-responsive polymer with biocompatibility and antibacterial activity, seem to be an ideal candidate to apply in drug delivery systems and other biomedical applications. These hybrid nanoparticles have been proven to have a high storage capacity that can be used in delivery system for medicines. The external pH-responsive PDMAEMA layer covered mesoporous silica materials allows to control release of encapsulated molecules [72].

**Scheme 15:**
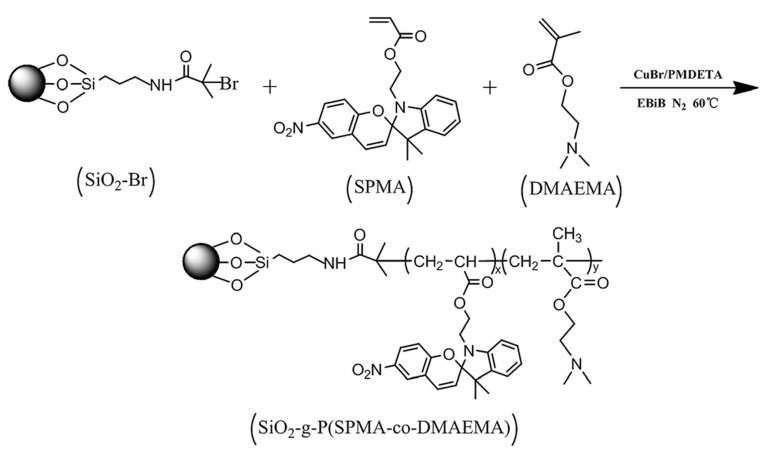
Schematic illustration of synthesis route of SiO_2_-*g*-P(SPMA-*co*-DMAEMA). Reproduced with permission from reference [73].

More recently, Liu et al. [73] have reported triple responsive polymer brushes including thermo-, pH- and photo-sensitive monomers (Scheme 15). The copolymer brushes of DMAEMA and 1’-(2-acryloxyethyl)-3’,3’-dimethyl-6-nitrospiro-(2H-1-benzopyran-2,2’-indoline) (SPMA) were synthesized from 2-bromoisobutyrate-functionalized Si NPs *via* SI-ATRP with 6260 ppm Cu catalytic complex. 

Palladium is usually used as a catalyst in the hydrogenation reaction of styrene. Catalytically active metal can be loaded into substrates of various types. The comparison of the catalytic action of SiO_2_-*g*-P(SPMA-*co*-DMAEMA) without loading palladium and SiO_2_-*g*-p(SPMA-*co*-DMAEMA)-Pd clearly indicates much better catalytic activity of hybrid silica after palladium loading [73].

**Scheme 16:**
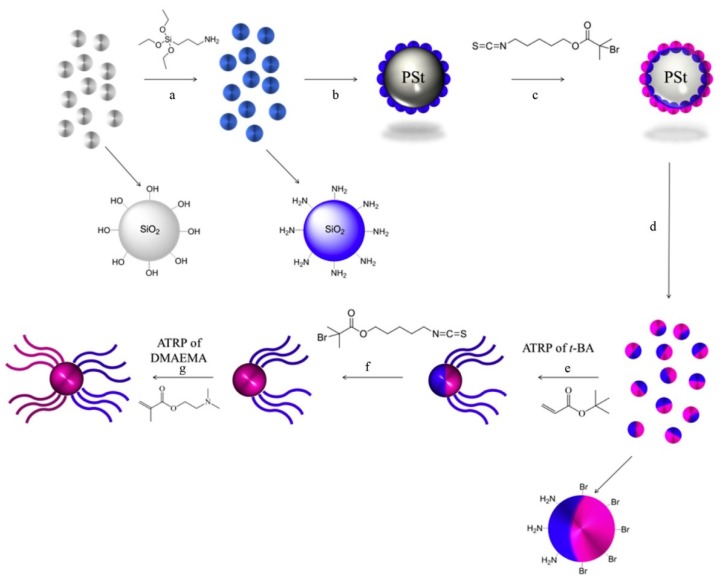
Schematic illustration of synthetic procedure followed for the preparation of the Janus silica NPs bearing P*t*BA and PDMAEMA polymer brushes on their two opposite hemispheres. Reproduced with permission from reference [75].

The original idea of pH-responsive polyampholytic hybrid Janus nanoparticles was presented by Falireas et al. [75]. Authors prepared hybrid nanoparticles comprising an inorganic silica core and a shell including poly(acrylic acid) (PAA) and PDMAEMA grafted polymer chains according to ATRP methodology with 210,000 ppm of Cu^I^Br/PMDETA complex. Janus NPs initiators were successfully synthesized using the Pickering emulsion method (Scheme 16). For this purpose, silica nanoparticles were previously functionalized with APTES and then used to stabilize PS molecules. Hybrid colloidosomes with a diameter of 1 mm were formed and the packing density of colloidal silica beads on the PS surface was quite low. Before the polymerization of DMAEMA from the uncovered surface of the P*t*BA-*g*-SiO_2_-*g*-APTES, it was necessary to eliminate the bromide end-groups of the polymer to avoid the chain growth of the P*t*BA chains with DMAEMA on the first hemisphere of the NPs. In accordance with literature data [96,97], bromide groups were removed by a radical chain transfer reaction using excess PMDETA in the presence of Cu^I^Br under monomer free conditions.

Finally, a well-defined hybrid of polyampholyte Janus NPs consisting of a metallic core and a polymeric shell from the PAA and PDMAEMA chains was synthesized using multistage surface initiated ATRP and post-polymerization polymer hydrolysis process.

## 4. Magnetic Nanoparticles (MNPs)—Iron Oxide Modified *via* SI-ATRP

Nanoparticles of metal oxides, especially iron oxides, show many distinctive properties, including electrical, optical and magnetic properties, which are not observed in the traditional form. In recent years they have been used in catalysis, photomagnetism, magnetooptics, drug delivery systems, medical diagnostics and cancer treatment [61,98]. Moreover, magnetic nanoparticles in the form of magnetite (Fe_3_O_4_) have unique properties that make them promising antimicrobial agents, especially that the US Food and Drug Administration (FDA) found that superparamagnetic nanoparticles iron oxide (SPIONP) is biocompatible (BC) with the human body [99].

Recent development in the field of the modification of magnetic nanoparticles, in particular the use of RDRP [34,61,98,100,101,102,103,104], have led to the invention of recycling systems to ensure oil and water separation [98] or potential MRI contrast agent for preclinical and clinical imaging [103]. Such a wide variety of application potential of iron oxide nanoparticles with grafted polymer layers results in a constant interest of researchers, therefore, selected papers of the last decade on the above-mentioned topics will be presented in this subchapter.

**Scheme 17:**
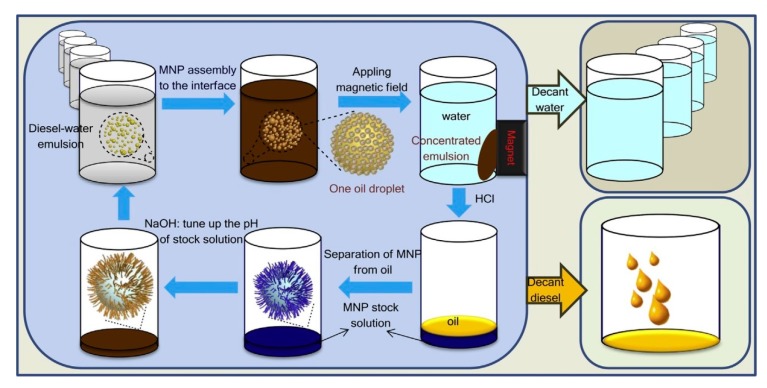
Application of pH-responsive magnetic nanoparticles (MNPs) as recyclable stabilizers for oil–water separation. Reproduced with permission from reference [98].

Wang et al. [98] have developed pH-responsive magnetic nanoparticles for oil-water separation (Scheme 17). Well-defined recyclable stabilizers with core-shell structure consist of Fe_3_O_4_ magnetic core and PDMAEMA organic shell showed that MNPs with longer PDMAEMA arms exhibited broader suitable pH range to form Pickering emulsion, but slower magnetic responsiveness. 

It is expected that this new conception of hybrid MNPs will prove to be a convenient and environmentally friendly way to separation of water contaminated with oil.

Another interesting example of using the magnetic properties of iron oxide nanoparticles modified with SI-ATRP is magnetic sorting of macrophages [61].

**Scheme 18:**
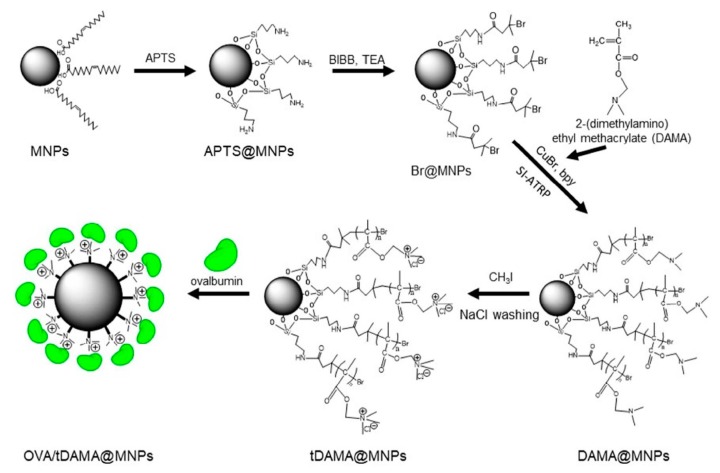
Schematic illustration of surface-decoration of MNP to prepare *t*DAMA@MNPs for electrostatic adsorption of OVA. Reproduced with permission from reference [61].

To decorate MNPs with a cationic corona, at the beginning the bromine initiator was attached to the surface of the MNPs, and then the surface was modified with PDAMA by SI-ATRP (with 10,000 ppm of Cu^I^Br/bpy) (Scheme 18). Subsequently tri-methylation greatly contributed to increase antigen payload on nanoparticles.

Macrophages incorporating ovalbumin (OVA)-loaded MNP was selected in magnetic fields and the cell population proved higher production of pro-inflammatory cytokines. This suggests that OVA-loaded nanoparticles can potentially increase the efficiency of immune therapy during the antigen-presenting pathway.

**Scheme 19:**
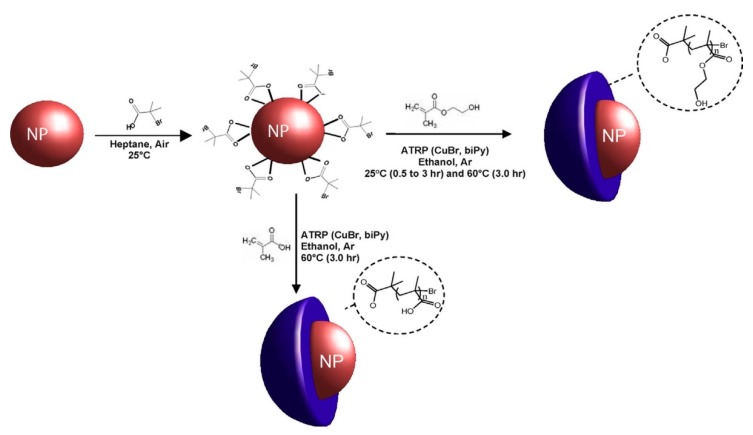
Schematic illustration of NPs surface functionalization by PHEMA and PMAA polymer brushes. Reproduced with permission from reference [34].

Recently, poly(hydroxyethylmethacrylate) (PHEMA) and poly(methacrylic acid) (PMAA) were grafted from γ-Fe_2_O_3_ NPs according to Scheme 19 [34]. Superparamagnetic maghemite covered with different hydrophilic polymer layers was synthesized using surface initiated atomic transfer radical polymerization (SI-ATRP), leading to stable colloids in water. Ten-nm sized γ-Fe_2_O_3_ iron oxide NPs were modified using Cu^I^Br/bpy as a catalyst (5100 and 3540 ppm of Cu species for HEMA and MAA, respectively). These synthesized nanomaterials provide biocompatible intelligent multifunctional nano-tools for magnetic resonance imaging (MRI), magnetic hyperthermia and also magnetically stimulated drug release systems. 

More recently, novel water-dispersible hybrid iron oxide nanoparticles grafted with a polymeric analogue of dimethylsulfoxide (DMSO) were developed as a potential MRI contrast agents [103]. 

Superparamagnetic iron oxide with immobilized ATRP initiator was prepared by in situ method using 12-(2-bromoisobutyramido) dodecanoic acid (BiBADA). Then, the initiators for continuous activator regeneration atom transfer radical polymerization (ICAR) ATRP technique with 390 ppm of Cu^II^Br_2_/Me_6_TREN complex was applied to grafted poly(2-(methylsulfinyl)ethylacrylate) (PMSEA, a polyacrylate analogue of DMSO) brushes from nanoparticle surface [103]. A study on the biocompatibility of this new hybrid material confirmed the non-toxicity of molecules over a wide range of concentrations. Therefore, already small doses of newly developed nanoparticles are effective as non-toxic MRI contrast agents [103].

**Scheme 20:**
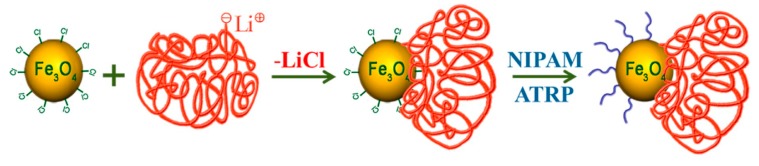
Schematic illustration of synthesis of a polymer−Fe_3_O_4_ composite Janus NP by grafting single chains. Reproduced with permission from reference [101].

In 2018, polymer-Fe_3_O_4_ composite Janus nanoparticles were synthesized [101] to achieved a nanomaterial with promising thermal and magnetic properties. Scheme 20 depicts the synthesis of a polymer−Fe_3_O_4_ composite Janus NP by grafting a single chain. At first, chloromethylphenyl-group was prepared by ligand exchange with the hydrolyzed silane from the oleic acid capped NP. Following, a single chain of anionic living polystyrene was grafted onto the NPs surface by a rapid elimination of Cl from the NPs surface with Li anion of the polymer. Finally, thermo-responsive PNIPM brushes were grafted from the opposite side of NPs by SI-ATRP.

Shape and stability drops of emulsion of the obtained magnetomaterial can be manipulated in the magnetic field. As a result of the photo-thermal action of Fe_3_O_4_ NPs at NIR irradiation, the destabilization of the emulsion is caused by NIR irradiation at low ambient temperature. Hyperthermia of Fe_3_O_4_ NPs and fast transfer of thermal energy used to heating PNIPAM above LCST is the reason of observed interactive reaction of NPs [101].

## 5. Other Nanoparticles Modified with SI-ATRP

Apart from typical nanoparticles, over the most popular such as silica nanoparticles or the magnetic nanoparticles described in this review, in the literature there are reports of successful ATRP synthesis of hybrid nanomaterials containing zinc [39,105], cerium [106] or aluminium [107], titanium oxide [55] as a metallic core of core–shell structure. Surface of superparamagnetic cobalt nanoparticles [56], graphene oxide [108], or boron nitride nanosheets [109] were successfully functionalized with the ATRP approach in order to apply it in the fields of biomedical and thermal management in electronics.

**Scheme 21:**
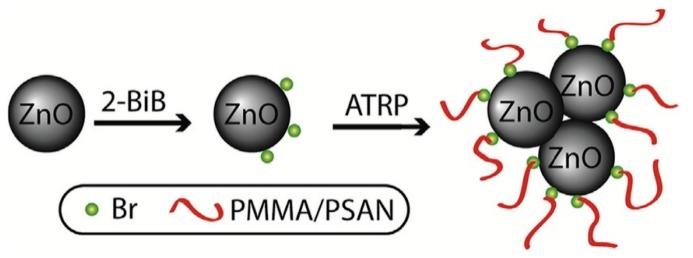
Schematic illustration of synthesis of polymer brush grafted ZnO NPs by “grafting-from” method. Reproduced with permission from reference [39].

Ding et al. [39] have focused on synthesis of polymer-tethered ZnO hybrid materials with three distinct approaches including among others “*grafting-from*” and “*grafting-onto*” methods. In “*grafting from*” approach, the initial stage of synthesis was to functionalize the surface of nanoparticles by attaching 2-BIB (Scheme 21). The (co)polymer brushes (PMMA or PMMA/P(S-*co*-AN)) were grafted from the surface of initiator-modified zinc oxide nanoparticles using Cu^II^Br_2_/Me_6_TREN as a catalyst complex (120 ppm) [39]. 

The “*grafting-onto*” method was based on PMMA/PSAN-*b*-PAA adsorption on the surface of the nanoparticle metallic core (50 and 200 ppm of Cu^II^Br_2_/Me_6_TREN for MMA and *t*BA polymerizations, respectively). 

Both ATRP synthetic approaches resulted in ZnO NPs easily dispersed in organic solvents. However, gradual aggregation of nanoparticles in the solution indicates low grafting density of polymer brushes, which limited stabilization of the obtained nanoparticles [39]. 

Another interesting example of NPs modified by SI-ATRP with ppm of Cu^I^Br/1,1,4,7,10,10-hexamethyltriethylenetetramine (HMTETA) complex is novel nanocomposites of poly(laurylmethacrylate)-grafted Al_2_O_3_ nanoparticles described by Sanchez et al. [107]. Authors synthesized nanomaterial by mixing the grafted NPs in an LDPE matrix in different ratios. As a result, obtained nanocomposite can be used in the production of newly designed dielectric materials with enhanced mechanical properties even at small amounts of inorganic filler. 

Meanwhile Wu et al. [109] presented an unconventional idea to modify boron nitride nanosheets (BNNSs) by polyelectrolytes *via* activators regenerated by electron transfer atom transfer radical polymerization (ARGET) ATRP with 4270 ppm of Cu^I^Br/PMDETA catalyst approach was applied to grafted brushes of a typical polyelectrolyte such as poly 2-acrylamido-2-methylpropanesulfonate (PAMPS). Traditionally, a two-step “*grafting from*” approach has been applied, initially with the use of BiBB to functionalize the surface, followed by grafting of PAMPS brushes from BNNSs surfaces. 

The layer of polyelectronlitic brushes synthesized on surface of BNNSs increased its dispersion in aqueous solvent, therefore authors expect that highly dispersed functional BNNSs/PAMPS composites can be used in biomedicine and thermal management in electronics [109].

## 6. Cellulose Nanocrystal Modified *via* SI-ATRP—Nature-Based Nanomaterials

In recent years, nanocellulose has been hailed as a wonderful material with flexibility and tensile strength greater than carbon fibers and steel [110,111]. Biodegradable raw material with an extremely low cost has proved to be a promising a natural material with high application potential. The nanocellulose crystals (CNC) issue has resulted in the publication of numerous papers presenting the effect of CNC modification using controlled radical polymerization in the last decade [112,113,114,115,116,117,118,119,120]. The use of the ATRP technique for a material that is CNC has enabled the control of the structure of grafted polymer chains leading to the acquisition of a well-defined nature-based nanomaterial.

**Scheme 22:**
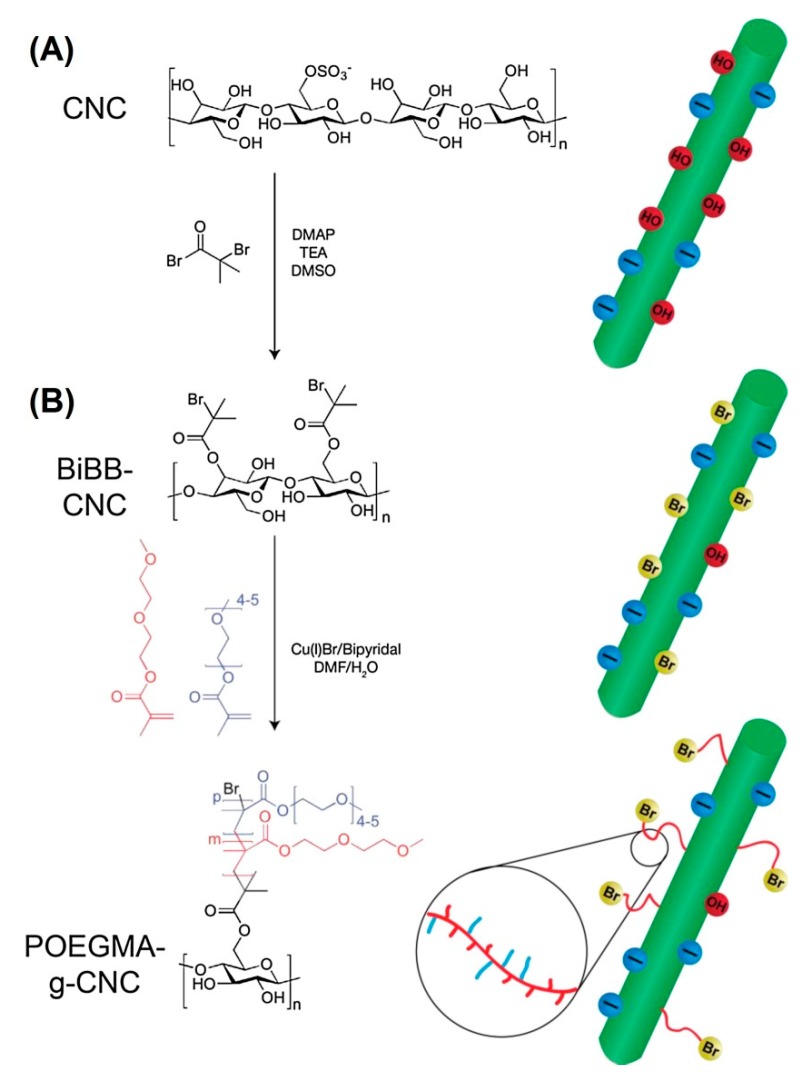
Synthesis of cellulose nanocrystal-*graft*-poly(oligo(ethylene glycol) methacrylate) (CNC-*g*-POEGMA) (**A**) synthesis of macro-initiator CNC (**B**) grafting of MEO_2_MA:OEGMA_300_ onto surface of modified CNC. Reproduced with permission from reference [113].

Grishkewich et al. [113] modified the CNC surface with thermo-sensitive brushes of two analogues of poly(oligoethylene glycol) methyl ether acrylate (POEGA) through SI-ATRP starting from the covalent connection of BiBB acting as the initiator of the polymerization process (Scheme 22). Copolymerization of di(ethylene glycol) methyl ether methacrylate (MEO_2_MA) and oligoethylene glycol methyl ether methacrylate (OEGMA_300_) monomers from the surface in the presence of Cu^I^Br/bpy catalytic complex (80,000 ppm) leads to a hybrid material with differentiated LCSTs. 

Depending on the ratio of thermo-responsive monomers used for the synthesis of nanomaterial based on CNCs, the LCSTs values in aqueous medium can be adjusted [113]. In the CNC-*g*-POEGMA system, the content of MEO_2_MA and OEGMA_300_ affects the LCSTs value between 23.8 and 63.8 °C. Microstructural analysis of the material showed that below LCSTs, the polymer chains collapse into hydrophobic globules on the surface of CNCs, thus inducing the aggregation of nanoparticles [113]. Moreover, as a result of heating and cooling of the sample by means of cloud point measurements, hysteresis of the tested material was recorded. This hysteresis is attributed to an intramolecular hydrogen bond between the collapsed POEGMA chains and hydroxyl groups on cellulose nanocrystals [113].

Recently, modification of cellulose nanocrystal *via* SI-ATRP of styrene and the mechanism of its reinforcement of PMMA was presented [114]. The aim of the modification was to synthesize a bionanofiller for the production of nanocomposites with improved thermal and mechanical properties.

The addition of 1% CNC to PMMA and production of CNC/PMMA composite improves the strength to break and increases elongation at break, however, due to CNC aggregation the mechanical properties of the material deteriorate. Interestingly, addition of more than 1% of filler causes a decrease in tensile strength resulting from the concentration of stresses resulting from CNC aggregation. However, the comparison of elongation at break and tensile strength of PMMA composites and pure CNC is weaker than pure PMMA [114]. Finally, as a result of an application of the filler modified with ATRP technique, thermal stability, breaking strength and elongation at break of the PMMA/CNC composites were improved, and a composite with excellent transparency has been obtained.

**Scheme 23:**
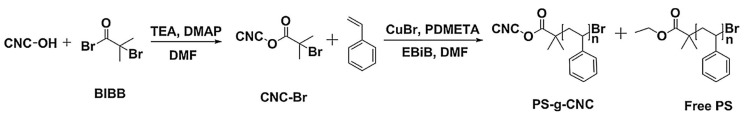
Synthetic route used for the preparation of the PS-g-CNC and free PS. Reproduced with permission from reference [118].

In turn, Zhang et al. [118] reported on synthesis (according to Scheme 23) and developed a convenient method to characterize the grafted PS on the CNC surface by SI-ATRP (2000 ppm of Cu species) without cleaving the grafted polymers. SI-ATRP synthesis was carried out in DMF solvent in the presence of EBIB as a sacrificial initiator and the brominated CNC nano-initiator (CNC-Br) previously synthesized by CNC esterification with EBIB.

**Scheme 24:**
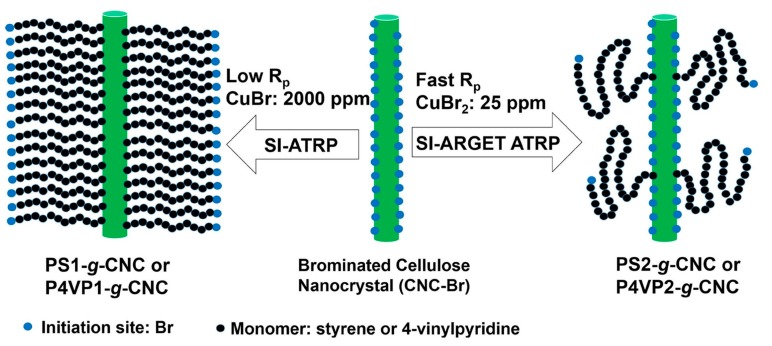
Schematic illustration of the proposed mechanism to account for the differences found with SI-ATRP and SI-ARGET ATRP from CNC-Br. Reproduced with permission from reference [120].

More recently, the comparative study on grafting polymers from cellulose nanocrystals *via* SI-ATRP and SI-ARGET ATRP was performed with the use of brominated macroinitiator and EBIB as the sacrificial initiator (Scheme 24). In the syntheses a 500-fold excess of monomer in relation to the initiator was applied to achieve high MW for grafted polymer brushes. The ARGET ATRP reaction was carried out in the presence of excess ascorbic acid as a reducing agent to ensure the continued regeneration of Cu^I^ of Cu^II^. Therefore, the amount of Cu catalyst was reduced from 2000 ppm in ATRP to 25 ppm in ARGET ATRP [120]. 

The differences found with SI-ATRP and SI-ARGET ATRP from CNC-Br showed that the latter one promotes the grafting of longer polymer chains with lower grafting densities. Observed differences between applied approaches resulted from significantly lower catalyst concentration (25 ppm) and higher propagation in the SI-ARGET ATRP system [120]. 

Meanwhile, Morits et al. [116] used controlled SI-ATRP for grafting polymer brushes onto a more complex and partially disordered form of nanocellulose, namely nanofibres cellulose (CNFs). CNFs modification was carried out by grafting poly(*n*-butyl acrylate) and PDMAEMA brushes *via* SI-ATRP with 260 ppm of Cu^II^Br_2_/PMDETA catalytic complex. For both reaction first order kinetics indicated controlled polymerization up to chain lengths of DP = 800. Depending on the number of initiation sites on the surface of nanofibres, the differences in cellulose backbone behavior were observed. Interestingly, high density of grafted polymer brushes led to degradation of nanofibre backbone, while for brushes with moderate and lower grafting density the integrity of nanofibre structure was preserved [116].

## 7. Summary

As shown in this review paper, the continuous search for modern methods of obtaining well-defined organic–inorganic hybrids with a core–shell structure is an extremely important challenge with many application possibilities. The most frequently modified nanoparticles include Au, Si or magnetic (mainly iron oxides) described in more detail. The use of the SI-ATRP technique allowed for the synthesis of materials with predefined structure and predetermined properties, which enables to design innovative nanomaterials responding to the needs of modern nanotechnology or biomedicine. Nowadays, the rapid development of these research area offers a wide range of possibilities for the application of the ATRP approach. The new ARTP procedures with reduced concentration of the copper catalytic complex make this technique an even more attractive and environmentally friendly tool for the synthesis of precisely defined nanomaterials. The indicated examples of SI-ATRP application show its leading application in the synthesis of stimuli-responsive materials used e.g., drug delivery systems or temperature sensors used in microelectronics devices.
